# The effect of sacrificing the posterior cruciate ligament on total knee arthroplasty with cruciate retaining highly congruent rotating platform prosthesis

**DOI:** 10.1186/s13018-021-02433-2

**Published:** 2021-05-07

**Authors:** Long Chen, Jie Xu, Yuan Lin, Fen Qi Luo, Yu Guo Yu

**Affiliations:** 1 Fujian Medical University, Fuzhou, 350000 China; 2 Department of Orthopedics, Fujian Provincial Hospital, Fuzhou, 350000 China

**Keywords:** Total knee arthroplasty, Cruciate retaining, Rotating platform, Highly congruent

## Abstract

**Objective:**

To analyze the effect of sacrificing the posterior cruciate ligament (PCL) on the early postoperative outcome of cruciate retaining (CR) highly congruent rotating platform TKA.

**Methods:**

From May 2018 to September 2019, 105 cases of total knee arthroplasty (TKA) with CR highly congruent rotating platform prosthesis were retrospectively analyzed. According to the tension of posterior cruciate ligament, they were divided into sacrifice group (29 cases, 27.6%) and retention group (76 cases, 72.4%). Preoperative and postoperative The Hospital for Special Surgery (HSS) score, range of motion (ROM) were compared between the two groups. In addition, postoperative infection, prosthesis loosening, bearing dislocation, and other complications were also compared.

**Results:**

All patients were followed up for 11~24 months (mean 18.14 ± 3.52) months. There was no significant difference in general data, preoperative HSS score, and ROM between the two groups (*P* > 0.05). At the last follow-up, HSS score and ROM of the two groups were better than those before operation (*P* < 0.05). However, there was no significant difference between the two groups (*P* > 0.05). Moreover, there were no complications such as infection, loosening of prosthesis, and bearing dislocation in all cases.

**Conclusion:**

In CR, highly congruent rotating platform TKA with or without tension of the PCL can achieve satisfactory outcomes. Tension-free PCL do not cause joint instability.

## Background

Total knee arthroplasty is the first choice for the treatment of end-stage knee osteoarthritis. A number of studies have been conducted on the comparison of posterior cruciate ligament (PCL) retention or sacrifice in total knee arthroplasty (TKA), but no difference in clinical outcomes has been found. In the cruciate retaining (CR) highly congruent rotating platform TKA, the excessive tension of the PCL and the tight flexion gap can easily lead to the bearing dislocation [1]. In this case, the PCL needs to be released as to reduce the tension. We found that although the PCL completely lost tension, this did not affect the stability of the prosthesis. Benjamin M. Stronach [2] reported that sacrificing the PCL did not cause dysfunction of the CR highly congruent fixed platform prosthesis. However, it has not been reported the effect of sacrificing the PCL on the knee function after TKA with CR highly congruent rotating platform prosthesis, which will be studied in this study.

## Methods

### General data

From May 2018 to September 2019, 105 cases of TKA with CR highly congruent rotating platform prosthesis were retrospectively analyzed. Inclusive criteria: (1) primary TKA; (2) CR highly congruent rotating platform prosthesis (Johnson, USA); (3) the patients were followed up for more than 3 months. Exclusion criteria: the follow-up time was less than 3 months. There were 7 cases of rheumatoid arthritis, 95 cases of osteoarthritis and 3 cases of traumatic arthritis. There were 26 males and 79 females. The average age was 69.22 ± 8.86 years (48–91 years). According to the continuity of PCL and tension after release, the patients were divided into sacrifice group (29 cases, 27.6%) and retention group (76 cases, 72.4%). (The tension of the PCL was mainly judged by the surgeon through observing and touching the PCL.) There was no significant difference in general data (age, weight, BMI, etc.) between the two groups (Table 1).

**Table 1:** Comparison of preoperative general data between the two groups

Group	Number	Age	Gender		Weight	BMI
		(*x* ± s, year)	Male	Female	(*x* ± *s*, kg)	(*x* ± *s*, kg/cm^2^)
Sacrifice group	29	68.97 ± 9.30	9	20	65.69 ± 7.71	26.37 ± 3.57
Retention group	76	69.39 ± 8.65	17	59	65.44 ± 10.4	25.22 ± 3.95
*P* value		0.857			0.930	0.203

### Surgical intervention

TKA was performed by the same senior orthopedic surgeon in both groups. After general anesthesia, the patient was placed in supine position. and the thigh was pressurized with tourniquet. The knee joint was exposed without eversion of the patella through the medial femoral muscle approach [3]. Osteophytes were removed, anterior cruciate ligament (ACL) was cut off, and the continuity of PCL was evaluated. Intramedullary and extramedullary localization were used for distal femoral osteotomy and tibial plateau osteotomy respectively, and then the extension space was balanced. The balance of the flexion gap was achieved by the method of gap balance. Under the condition of fully tensioning the self-developed ranging balancer, the same distance as the extension gap was set for osteotomy of the posterior femoral condyle. Then the model test was carried out. In case of gasket lift off, rotation off, or excessive roll back of femur, priority should be given to adjusting the posterior tibial slope and properly releasing the posterior cruciate ligament to make the above situation disappear. After all osteotomies were completed, the patella was semi turned in the extension position for patellar replacement. After the test piece was suitable, the prosthesis was installed, the incision was sutured, and no drainage tube was placed.

### Anesthesia choice

All patients were given general anesthesia, considering the following advantages: (1) general anesthesia passes through the natural cavity, reducing the invasive operation and possible complications caused by spinal anesthesia; (2) spinal anesthesia may fail in older patients with cone degeneration, affecting the surgical handover time. Therefore, we only chose spinal anesthesia for patients with severe cardiopulmonary diseases and intolerance of general anesthesia.

### Postoperative management

The patient walked on the ground after recovery from anesthesia, and took quadriceps exercise, active and passive knee flexion, and extension exercise. On the second day after the operation, they were trained to go up and down stairs and were given analgesic and anticoagulant treatment according to the conventional requirements.

### Follow-up and evaluation index

The data of gender, age, BMI, ROM, and HSS score were recorded before operation. The patients were followed up at 1, 3, 6, 12, 18, and 24 months after operation. The ROM and HSS scores (including pain, function, range of motion, muscle strength, flexion deformity, and stability) of knee joint were used for clinical evaluation, and anteroposterior and lateral X-ray of knee joint and full length X-ray were taken for imaging evaluation. In addition, postoperative complications such as infection, prosthesis loosening and osteolysis were also evaluated.

### Statistical analysis

The data were processed by SPSS19.0 statistical software. The measurement data were expressed as mean ± standard deviation (*x* ± *s*), and the comparison between groups was conducted by *t* test; the comparison of counting data between groups was performed by *χ*^2^ test. *P < 0.05* was defined as statistically significant.

## Results

**Fig. 1:**
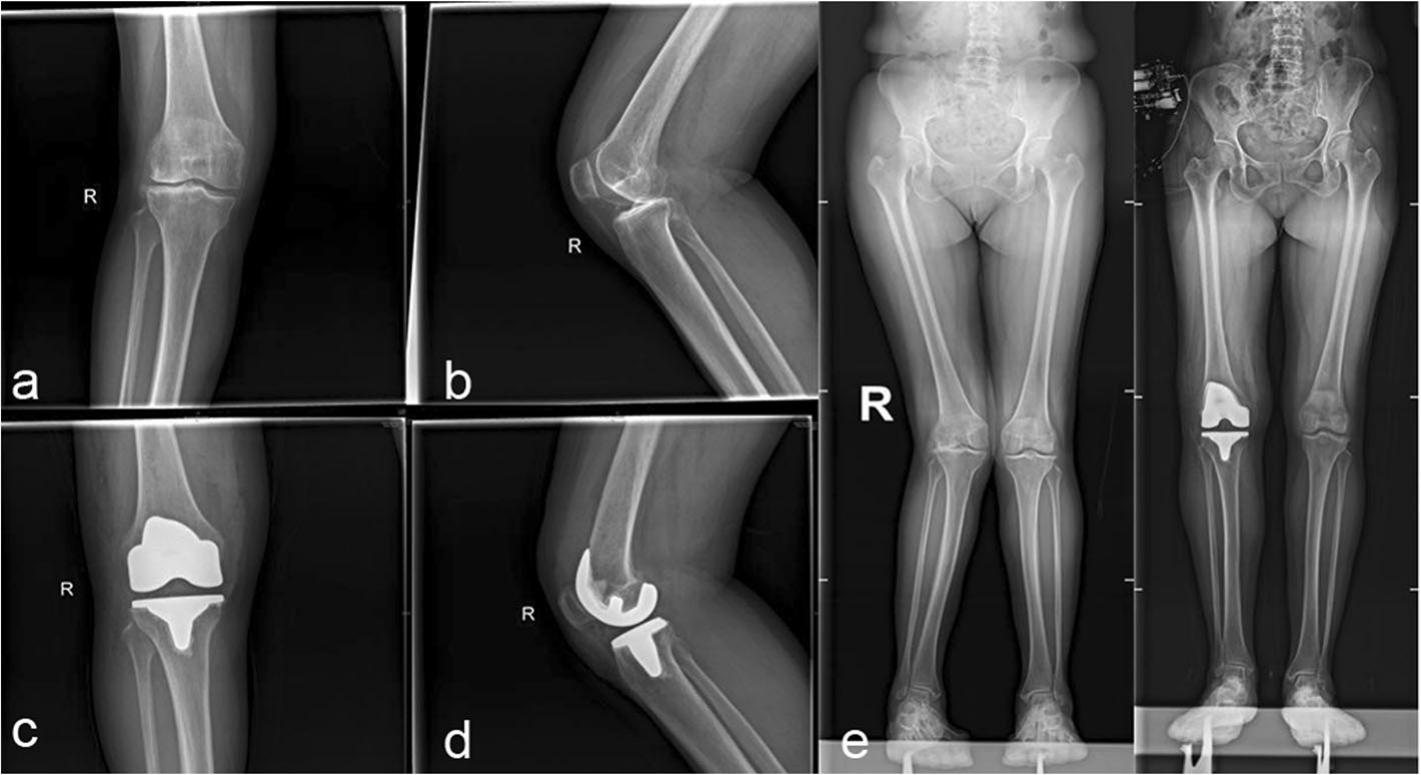
A 65-year-old female with osteoarthritis. **a**, **b** Preoperative anteroposterior and lateral X-ray showed that the narrow knee joint space and a large amount of hyperosteogeny. **e** Preoperative full length X-ray showed valgus deformity. **c**, **d** One year after operation, the anteroposterior and lateral X-ray showed that the prosthesis was in suitable position, and there was no light transmission zone at the interface between prosthesis and bone. **f** One year after the operation, full length X-ray showed correction of lower limb alignment

**Fig. 2:**
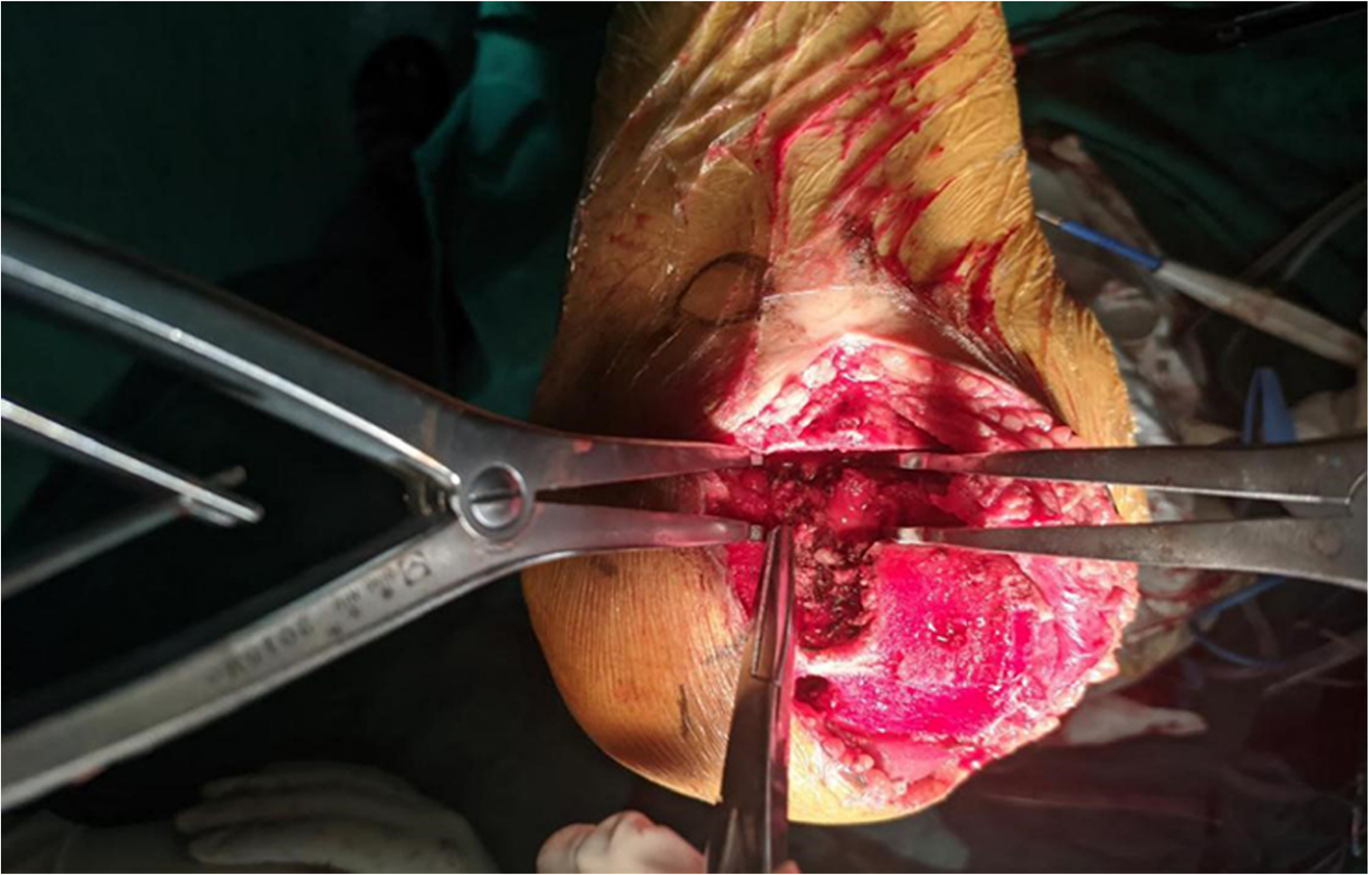
The continuity of posterior cruciate ligament is destroyed

All patients were followed up for 11~24 months (mean 18.14 ± 3.52) months. The average follow-up time was 19.4 ± 2.90 months in the sacrifice group and 17.7 ± 3.64 months in the retention group. The ROM and HSS scores of the two groups were significantly improved after operation (*P < 0.05*). However, there was no significant difference in ROM and HSS scores between the two groups (*P > 0.05*, Tables 2 and 3). There was no loosening and displacement of prosthesis, no postoperative complications such as joint infection, deep vein thrombosis, and joint dislocation in both groups. The image data of typical cases before and after operation are shown in Fig. 1. Intraoperative assessment revealed no tension in the posterior cruciate ligament (Fig. 2). (The device shown in the figure was similar to a spreader, which gradually increased the tension through the card slot to achieve a balance between the inner and outer tension and the flexion gap.)

**Table 2:** Comparison of preoperative and postoperative range of motion between the two groups

Group	Number	Range of motion			
		Preoperation	Postoperation	*t* value	*P* value
Sacrifice group	29	96.97 ± 8.30	126.00 ± 6.99	12.996	0.000
Retention group	76	95.79 ± 8.08	124.64 ± 7.48	22.949	0.000
*t value*	–	0.662	0.844	–	–
*P* value	–	0.510	0.400	–	–

**Table 3:** Comparison of preoperative and postoperative HSS scores between the two groups

Group	Number	HSS scores			
		Preoperation	Postoperation	*t* value	*P* value
Sacrifice group	29	55.31 ± 6.46	91.45 ± 4.21	23.716	0.000
Retention group	76	54.76 ± 6.34	90.43 ± 4.09	40.188	0.000
*t value*	–	0.393	1.127	–	–
*P* value	–	0.695	0.263	–	–

Among the 7 cases of RA, 3 cases had their PCL eroded to form scar tissue and without tension, but they could still be identified as PCL based on their location. The 3 cases were classified as the sacrifice group. In the other 4 cases, the PCL was well preserved without losing tension.

Among the three patients with traumatic arthritis, two cases were caused by anterior cruciate ligament injury and one case was caused by tibial plateau fractures. During the intraoperative exploration of PCL, the morphology was intact, undamaged, and good tension was maintained. The three cases were classified as the retention group.

## Discussion

The traditional CR prosthesis relies on the proprioception of the PCL and causes femoral roll back through ligament tension to achieve joint flexion and stability [4]. The traditional CR prosthesis needs to retain high function of the PCL. However, it may be found that the PCL has lost tension long or after release during TKA, which leads to the instability of the prosthesis. In addition, in order to obtain excellent knee flexion and avoid restricting the rolling motion of the femur, the traditional CR prosthesis uses flat gasket. Wada et al. [5] analyzed the kinematics of four types of prostheses in TKA and pointed out that the prostheses with flat gaskets were prone to displacement and wear. Daniilidis et al. [6] found that compared with the flat gasket, the highly congruent gasket effectively reduced the contradictory forward displacement and non-physiological roll back, while the rotating platform avoids the limitation of the highly congruent front and rear lips on the femoral condyle roll back and tibial rotation during high flexion. At the same time, the lateral condyle roll back during flexion, which is more in line with physiological knee kinematics.

At present, numerous studies have reported that the use of highly congruent rotating platform prosthesis is conducive to joint stability [7]. Peter et al. [8] compared traditional CR prosthesis with highly congruent prosthesis and found that there was no difference between the two groups in terms of function score, complications, etc., but the revision rates of traditional CR prosthesis was higher, among which the instability of knee joint was the most crucial reason.

**Fig. 3:**
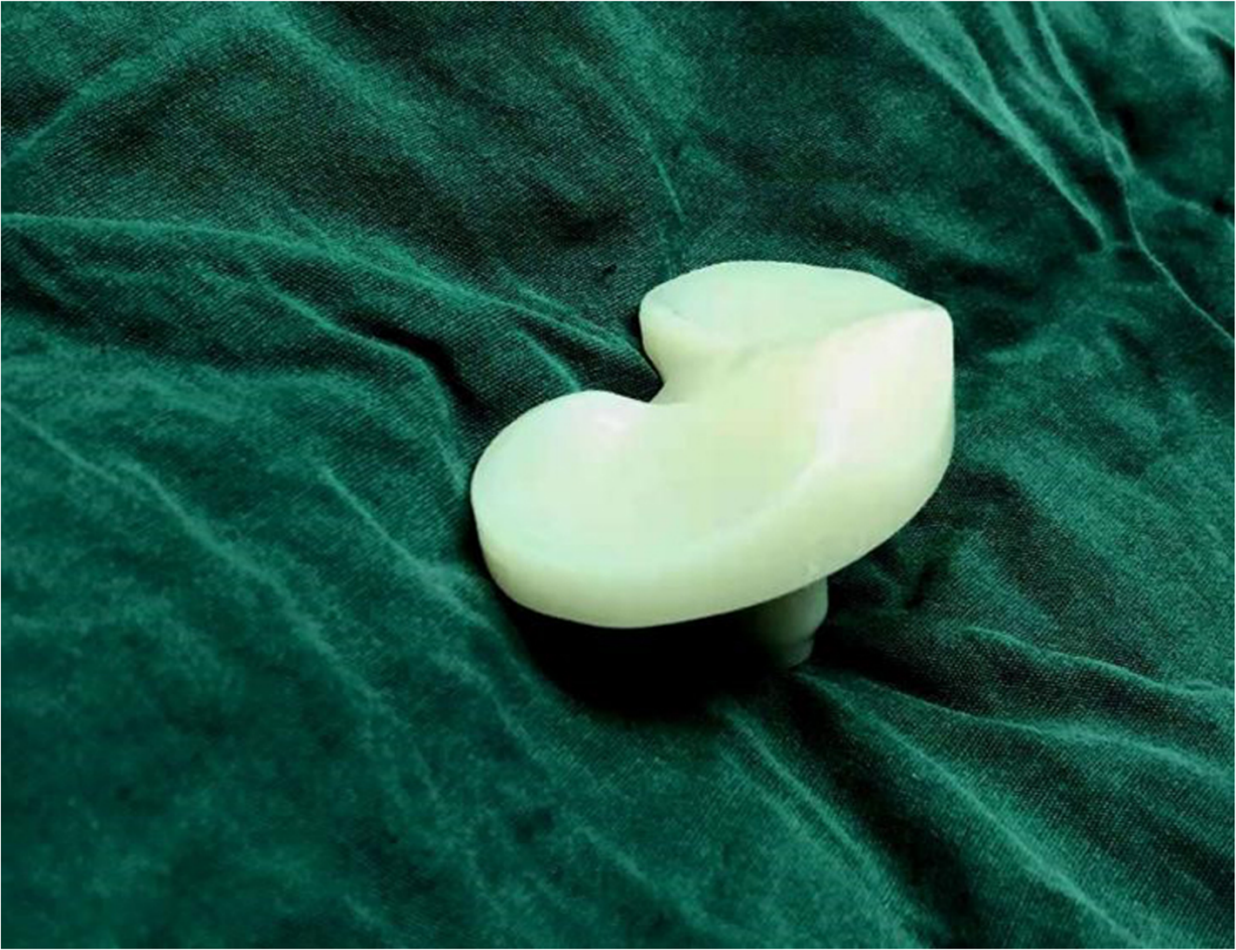
highly congruent gasket

Comparing the revision risk of the rotating platform TKA with that of the fixed platform TKA, Marek Lacko [9] proposed that the rotating platform TKA may increase the risk of revision risk at an early stage due to knee instability, mainly on account of improper surgical techniques, such as unbalance of flexor-extension gap and dislocation of components. In our study, neither sacrifice nor retain the PCL resulted in early revision due to joint instability, which is attributed to a suitable gap balance to improve joint stability. The deep disk design of the highly congruent gasket (Fig. 3) increases the contact area between the gasket and the femoral interface, which improves the congruent between the femoral component and the gasket. Even in the absence of the PCL, the protruded anterior lip of the gasket plays a role in limiting the excessive forward movement of the femoral condyle during flexion [10]. At the same time, the pressure of the patella prevents the femur from moving forward and causes it to roll backward. The combination of the above two items effectively avoids the occurrence of bearing dislocation when the knee joint is over-extended or over-flexion.

The highly congruent gasket improves the stability of the prosthesis and overcomes the requirement of high function of PCL. The results of this study are consistent with those of Benjamin M. The congruence of the rotating platform is higher than that of the fixed platform. Therefore, in the TKA of the rotating platform with highly congruent, if the tension of the posterior cruciate ligament is lost during the operation, as long as the medial and lateral collateral ligaments are well balanced, the joint range of motion and stability will not be affected.

It should be noted that in patients without developmental articular deformity due to wear and tear, parallel flexion and extension gap can be obtained after the removal of osteophytes and osteotomies without the need for release or extension of the medial and lateral ligaments. In such patients, the coordination of the cruciate ligament and the collateral ligament is synchronous. Therefore, as long as the proper posterior tibial slope is ensured, postoperatively the tension of the posterior cruciate ligament is usually not higher than that of the collateral ligament, that is, the excessive backward rolling of the femur, and the release of the posterior cruciate ligament is not required. However, for patients with developmental extraarticular deformity, the medial collateral ligament may have a higher tension than the lateral collateral ligament after osteotomy. It is required that the tension on both sides of the gasket must be equal; otherwise, the gasket will prolapse and wear will be accelerated. In this case, it is necessary to release and extend the ligament on the tight side. In addition, after placing the gasket matching the length of the collateral ligament, if the posterior tibial slope is unsuitable, the tension of the PCL will exceed that of the collateral ligament, which causing the bearing dislocation. In the early stage, we usually attributed the tight flexion space to insufficient release of the posterior cruciate ligament, rather than the posterior tibial slope. At the same time, it was not considered to distinguish the origin of varus and varus deformity, so we released the PCL resulting in PCL tension-free cases mostly occurred in the early studies. Then, we use the gap balance method to balance the flexion gap. In the case of a fully tensioned balancer, the gasket thickness should be reduced to obtain the appropriate buckling clearance. If there is still spacer prolapse at this time, it is preferred to increase posterior tibial slope (usually 7°) and then to release the PCL.

Although sacrificing PCL would bring about a decrease in proprioception, there was no need to deliberately change the surgical style and gasket during the operation. At the same time, a large number of osteotomies of PS prostheses were avoided. During the follow-up, there was no significant difference in the patient’s function. We believed that the benefit was greater than the impact on proprioception.

The results of our study confirmed that postoperative HSS scores and knee ROM of the two groups were significantly improved compared with preoperative those, which indicated that both sacrifice and retention of PCL could improve knee function in CR highly congruent rotating platform TKA.

There are still some deficiencies in this study: the sample size is not large enough. PCL tension-free patients mostly exist in the early stage of the study. The follow-up time is not long enough, and long-term follow-up is beneficial to clarify the impact of PCL ligament sacrifice on survival of prosthesis. The tension of the PCL was mainly judged by the clinical experience of the surgeon, and there was a lack of precise objective indicators.

## Conclusions

To sum up, in CR highly congruent rotating platform TKA, regardless of the posterior cruciate ligament tension or not, a satisfactory postoperative effect can be achieved without causing joint instability, and there is no need to replace the gasket due to the intraoperative PCL posterior cruciate ligament loss.

## Data Availability

The data used to support the findings of this study are available from the corresponding author upon request.

## Abbreviations

PCL: Posterior cruciate ligament
CR: Cruciate retaining
TKA: Total knee arthroplasty
HSS: The Hospital for Special Surgery
ROM: Range of motion
